# Complete mitochondrial genome of Asiagomphus coreanus (Odonata: Gomphidae), which is endemic to South Korea

**DOI:** 10.1080/23802359.2022.2072246

**Published:** 2022-05-09

**Authors:** Jeong Sun Park, Min Jee Kim, Sung Soo Kim, Iksoo Kim

**Affiliations:** a College of Agriculture & Life Sciences, Chonnam National University, Gwangju, Republic of Korea; b Experiment and Analysis Division, Honam Regional Office, Animal and Plant Quarantine Agency, Gunsan, Republic of Korea; c Institute for East Asian Environment and Biology, Seoul, Republic of Korea

**Keywords:** Mitochondrial genome, *Asiagomphus coreanu*, Gomphidae

## Abstract

*Asiagomphus coreanus* (Doi & Okumura, 1937) belongs to the family Gomphidae in the order Odonata, and has been listed as an endemic species in South Korea. Here, we assembled its complete mitochondrial genome (mitogenome) which is 15,649 base pairs (bp) in length. The *A. coreanus* mitogeneome consists of a typical set of genes [13 protein-coding genes (PCGs), 2 ribosomal RNA (rRNA) genes, and 22 transfer RNA (tRNA) genes] and one major non-coding A + T-rich region which is 846 bp long. The gene arrangement of the species was identical to that of commonly found in the majority of the insects. Phylogenetic analyses using the concatenated sequences of 13 PCGs and two rRNA genes of the representative odonate mitogenomes by Bayesian inference method revealed that *A. coreanus* belongs to the Gomphidae family with a strong nodal support (Bayesian posterior probabilities = 1). Unlike previous phylogenetic analyses (with regards to suborder relationships) the suborder Anisozygoptera—which was represented by a single species, *Epiophlebia superstes—*was placed as the sister to Zygoptera.

The dragonfly, *Asiagomphus coreanus* Doi & Okumura, 1937 is classified under the Gomphidae family in the order Odonata, and is an endemic species rarely found in South Korea (Lee 2013). *A. coreanus* can be easily recognized based on a broad yellow stripe on abdominal segment VII (Bae and Lee 2012). Larvae burrow under particular habitats in lowland river areas where the substrates are composed of mud and sand (Chung et al. 2016). Adults live the waterside and nearby forests, except during mating period (Chung et al. 2016). In this study, we analyzed the complete mitochondrial genome (mitogenome) of *A. coreanus*.

An adult *A. coreanus* was captured at Dongmak-ri, Galmal-eup, Cheorwon-gun, Gangwon-do Province, South Korea (38°14′50″ N, 127°18′00″ E) on June 2019. All authors promise that we have no any unethical behaviors for the study. The collection of insect material was carried out in accordance with guidelines and regulations provided by the authors’ institutions, local, national, and international regulations. If we have any unethical and illegal behaviors, we will take all responsibilities. Two hind legs were used for DNA extraction. Leftover DNA and specimen were deposited at the Chonnam National University, Gwangju, Korea, under the accession number CNU11150 (Iksoo Kim, ikkim81@chonnam.ac.kr). To obtain the complete sequence of the mitogenome of *A. coreanus,* whole genome sequencing was performed using the MGISEQ-2000 sequencing platform (MGI Tech Co. Ltd, Shenzhen, China). Construction of the genome was conducted using MITObim ver. 1.9 (Hahn et al. 2013) by *de novo* assembly. In order to obtain the precise genome sequence, gap filling was performed using the conventional Sanger-based sequencing method. For phylogenetic analysis, nucleotide sequences of 13 protein-coding genes (PCGs) and two rRNA genes of 35 species of the order Odonata belonging to 12 families and three species of Ephemeroptera (outgroups) were downloaded from GenBank and aligned. The optimal relationship tree was prepared with a Bayesian inference (BI) method using MrBayes v. 3.2.2 (Ronquist et al. 2012), implemented in the CIPRES Portal ver. 3.1 (Miller et al. 2010). For this analysis, the GTR + Gamma + I model, which was determined using PartitionFinder 2 and the Greedy algorithm, was used (Lanfear et al. 2012, 2014, 2017).

The *A. coreanus* mitogenome is 15,649 bp in length, with a typical set of genes (2 rRNAs, 22 tRNAs, and 13 PCGs) and a major non-coding A + T-rich region (GenBank accession number MN812523). The mitogenome of *A. coreanus* is well within the size range found in other insects belonging to the order Odonata. Thirteen PCGs had the typical ATN start codon, and nine of the 13 PCGs had a complete stop codon; however, *COI, COII, COIII,* and *ND5* had incomplete (T-) stop codons. The gene arrangement was identical to that typically observed in majority of other insects (Cameron 2014). The A + T-rich region of *A. coreanus* was 846 bp. The A/T content of the whole mitogenome was 70.8%; however, it varied among the genes as follows: 86.4%, A + T-rich region; 74.3%, *lrRNA*; 71.2%, *srRNA*; 71.3%, tRNAs; and 69.4%, PCGs.

Both the suborders Zygoptera and Anisoptera were shown to be monophyletic with the highest node support [Bayesian posterior probabilities (BPP) = 1.00; Figure 1]. All superfamilies and families represented by multiple taxa in Odonata were consistently revealed as being monophyletic with the highest nodal supports. The sister relationship between Zygoptera and Anisozygoptera (BPP = 0.99) was unconventional (Rehn 2003; Davis et al. 2011; Kim et al. 2014), but recent mitogenome-based phylogenetic results consistently supported the sister relationship between these two suborders (Yong et al. 2016; Jeong et al. 2018; Kim et al. 2018). *A. coreanus* was placed as the sister to *Davidius lunatus* with the highest nodal support.

**Figure 1.**
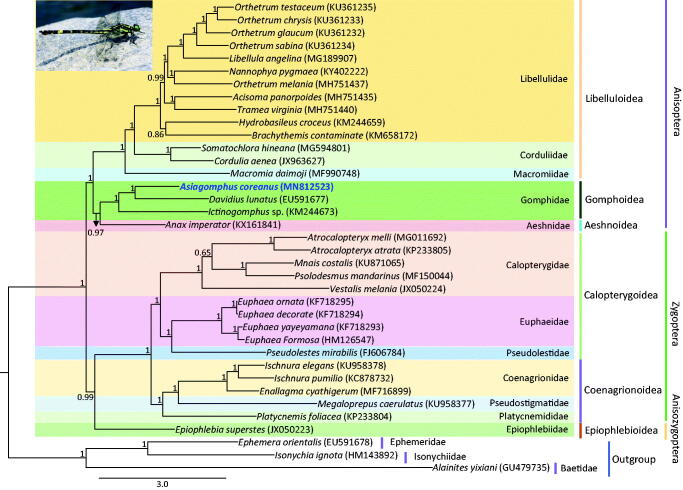
Phylogenetic tree for Odonata. The tree was constructed using the concatenated nucleotide sequences of 13 protein-coding genes (PCGs) and 2 rRNAs using the Bayesian inference (BI) method. The numbers at each node specify the Bayesian posterior probabilities (BPP). The scale bar indicates the number of substitutions per site. GenBank accession numbers of each species are shown in the brackets after scientific names.

## Data Availability

The genome sequence data that support the findings of this study are openly available in GenBank of NCBI at https://www.ncbi.nlm.nih.gov under the accession no. MN812523. The associated BioProject, SRA, and Bio-Sample numbers are PRJNA804682, SAMN25771832, and SRP359016, respectively.
